# ROS-ERK Pathway as Dual Mediators of Cellular Injury and Autophagy-Associated Adaptive Response in Urinary Protein-Irritated Renal Tubular Epithelial Cells

**DOI:** 10.1155/2021/6614848

**Published:** 2021-03-01

**Authors:** Jian-kun Deng, Xueqin Zhang, Hong-luan Wu, Yu Gan, Ling Ye, Huijuan Zheng, Zebing Zhu, Wei Jing Liu, Hua-feng Liu

**Affiliations:** ^1^Institute of Nephrology, Zhanjiang Key Laboratory of Prevention and Management of Chronic Kidney Disease, Guangdong Medical University, Zhanjiang, Guangdong 524001, China; ^2^Renal Research Institution of Beijing University of Chinese Medicine, and Key Laboratory of Chinese Internal Medicine of Ministry of Education and Beijing, Dongzhimen Hospital Affiliated to Beijing University of Chinese Medicine, Beijing 100700, China

## Abstract

ERK, an extracellular signal-regulated protein kinase, is involved in various biological responses, such as cell proliferation and differentiation, cell morphology maintenance, cytoskeletal construction, apoptosis, and canceration of cells. In this study, we focused on ERK pathway on cellular injury and autophagy-associated adaptive response in urinary protein-irritated renal tubular epithelial cells and explored the potential mechanisms underlying it. By using antioxidants N-acetylcysteine and catalase, we found that ERK pathway was activated by a reactive oxygen species- (ROS-) dependent mechanism after exposure to urinary proteins. What is more, ERK inhibitor U0126 could decrease the release of neutrophil gelatinase-associated lipocalin (NGAL), kidney injury molecule-1 (KIM-1), and the number of apoptotic cells induced by urinary proteins, indicating the damaging effects of ERK pathway in mediating cellular injury and apoptosis in HK-2 cells. Interestingly, we also found that the increased expression of microtubule-associated protein 1 light chain 3 (LC3)-II (a key marker of autophagy) and the decreased expression of p62 (autophagic substrate) induced by urinary proteins were reversed by U0126, suggesting autophagy was activated by ERK pathway. Furthermore, rapamycin reduced urinary protein-induced NGAL and KIM-1 secretion and cell growth inhibition, while chloroquine played the opposite effect, indicating that autophagy activation by ERK pathway was an adaptive response in the exposure to urinary proteins. Taken together, our results indicate that activated ROS-ERK pathway can induce cellular injury and in the meantime provide an autophagy-associated adaptive response in urinary protein-irritated renal tubular epithelial cells.

## 1. Introduction

Renal tubular epithelial cell injury is central to the pathophysiology of tubulointerstitial fibrosis, which strongly correlates with the decline of renal function [1–3]. Urinary protein, as a pathological product of glomerular diseases, is also an important factor leading to renal tubular epithelial cell injury. It is proved to cause cellular apoptosis, resulting in the detachment of renal tubular epithelial cells from the basement membrane and finally losing their functions to the renal tubules [4, 5]. Unfortunately, the underlying mechanisms of renal tubular epithelial injury induced by urinary protein remains unclear, limiting the research on effective therapy. Thus, it is of great importance to further explore the mechanism of urinary protein-induced cellular injury.

One of the important ways that urinary protein causes cellular injury is by elevating the production of reactive oxygen species (ROS) [6]. Moreover, ROS regulates various cellular signaling pathways, such as the ERK pathway [7]. With the phosphorylation of the Ras/Raf/MEK/ERK cascade, the ERK pathway is activated and participates in the regulation of a variety of cellular processes, which includes proliferation, transdifferentiation, autophagy, and apoptosis [8–10]. However, the role of ERK pathway in renal injury is still controversial until now. The ERK pathway has proved to be a pathogenic factor in various disease models, such as cystic kidney diseases [11], kidney stones [12], and unilateral ureter obstruction kidneys [13, 14]. More directly, Reich et al. indicated that the ERK pathway mediates the albumin-induced toxicity of TECs [15]. In contrast, Takase et al. demonstrated that the ERK pathway could serve as an important prosurvival factor to TECs under protein overload [16]. And recent studies verified that ERK pathway-mediated autophagy was protective in podocyte damage caused by lipopolysaccharide [17]. Given the wide range functions of ERK pathway, we hypothesized that it may play different roles simultaneously under the same condition. Hence, the aim of the study is to determine the exact role of ERK pathway in renal tubular epithelial cells irritated by urinary protein and provide a theoretical basis for the treatment of renal injury caused by urinary proteins.

## 2. Materials and Methods

### 2.1. Extraction of Urinary Proteins

Ammonium sulfate precipitation method as described previously [18] was used to extract the urinary proteins from untreated patients pathologically diagnosed as minimal change nephrotic syndrome (MCNS).

### 2.2. Cell Culture and Treatments

Human proximal tubular HK-2 cells (ATCC, Manassas, VA) were maintained in Dulbecco's modified Eagle's medium (DMEM, Gibco, Grand Island, NY) supplemented with 10% fetal bovine serum (Gibco) under standard conditions. The cells were pretreated with 10 mM N-acetylcysteine (NAC) (Beyotime Institute of Biotechnology, Jiangsu), 2000 U/ml catalase (Beyotime Institute of Biotechnology, Jiangsu), 10 *μ*M U0126 (Sigma, St. Louis, MO), 10 *μ*M rapamycin (Calbiochem, La Jolla, CA, USA), and 10 *μ*M chloroquine (Sigma) before the addition of urinary proteins (8 g/l). The cellular ROS production was measured at 2 h, and the expression of LC3 and P62 was quantified at 8 h. The levels of neutrophil gelatinase-associated lipocalin (NGAL) and kidney injury molecule-1 (KIM-1) secretion were tested at 12 h with the Quantikine™ kits (R&D Systems, Minneapolis, Minnesota, USA). The number of apoptotic cells was assayed at 48 h. And the expression of p-ERK and t-ERK was quantified at different time points.

### 2.3. Flow Cytometry Analysis

Cell apoptosis determination and cellular ROS production were measured with flow cytometry. All cells under various experimental conditions were harvested at 48 h with culture solution containing 0.05 trypsin and rinsed with PBS. Apoptosis was determined by the AnnexinV-FITC Apoptosis Detection Kit (Dojindo, Kumamoto, Japan) following the manufacturer's protocol with FACS Calibur flow cytometer (BD, FACSCanto II, San Jose, CA). For ROS measurement, HK-2 cells were incubated with DMEM containing 10 *μ*M 2′-7′-Dichlorodihydrofluorescein diacetate (DCFH-DA, Beyotime Institute of Biotechnology, Jiangsu) for 30 min in the dark. Then, harvested cells were trypsinized and resuspended in PBS. The fluorescence was determined by FACS Calibur flow cytometer (BD, FACSCanto II, San Jose, CA).

### 2.4. Western Blot

Autophagy response and ERK pathway were both examined by Western blot. At the end of incubation, cells were washed with PBS and lysed on ice for 15 min in 1× RIPA lysis buffer. Conditioned media and cell lysates were centrifuged at 13,000 rpm at 4°C for 15 min to pellet cell debris, and the concentration of cellular protein was determined using BCA reagent. Samples with equal concentrations of cellular protein were mixed with a 5× sample buffer and heated at 95°C for 10 min and separated on 12% SDS-PAGE gels. The samples were then transferred to a polyvinylidene difluoride membrane (Millipore, Billerica, MA, USA). After blocking with 5% fat-free milk for 2 h, the membranes were incubated overnight at 4°C with rabbit antibodies raised against microtubule-associated protein 1 light chain 3 (LC3)-II (Sigma, St. Louis, MO), p62/SQSTM1(Santa Cruz Biotechnology, Santa Cruz, CA), p-ERK (Cell Signaling Technology, Danvers, MA), and t-ERK (Cell Signaling Technology, Danvers, MA). After washing, the membranes were probed with HRP-conjugated secondary antibodies (Beyotime Institute of Biotechnology, Jiangsu) for 1 hour at room temperature. Immunoreactive bands were visualized using ECL plus Western blotting detection system (Pierce, Rockford, IL).

### 2.5. Cell Viability Assay

Cells were incubated with 5 mg/ml methyl thiazolyl tetrazolium (MTT) solution (Calbiochem) for 4 h at 37°C. The formazan crystals were dissolved in dimethylsulfoxide. Optical density was determined at 570 nm with a plate reader (Thermo Labsystems, Multiskan MK3, Shanghai, China).

### 2.6. Statistical Analysis

All statistical tests were performed with SPSS 16.0. Shapiro-Wilk test was used to detect the normality of variables. All data are expressed as the means ± standard error of the mean (S.E.M.). Multiple group comparison was carried out using ANOVA, followed by Bonferroni post hoc tests. *P* value was considered as statistically significant if it was less than 0.05.

## 3. Results

### 3.1. ERK Pathway in TECs Was Activated by Oxidative Stress after Exposure to Urinary Proteins

**Figure 1:**
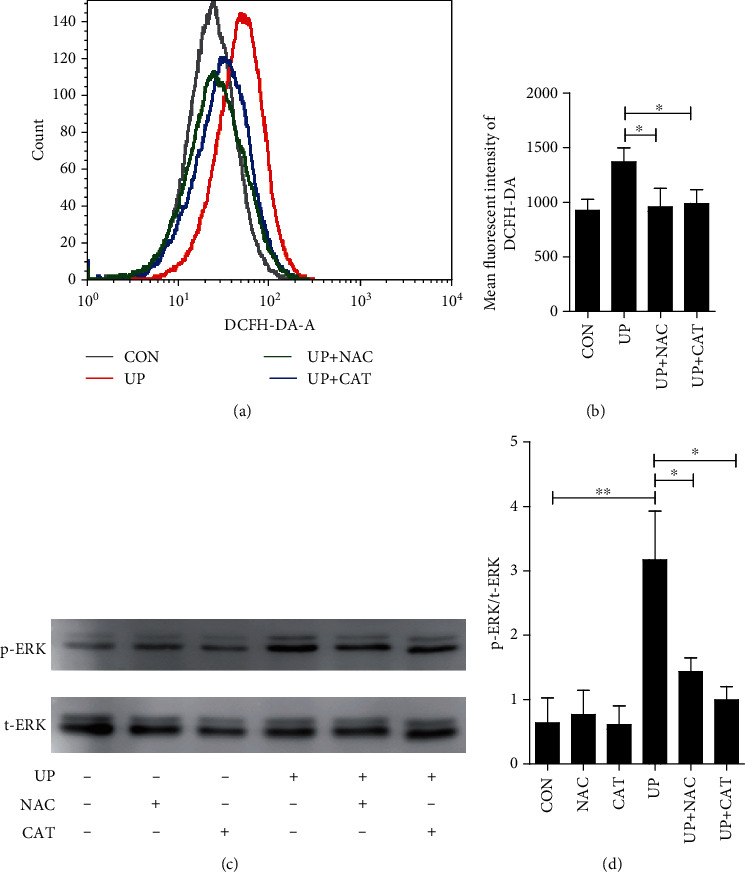
Effect of antioxidants on urinary protein-induced ERK pathway activation. Antioxidants NAC (10 mM) and CAT (2000 U/ml) were administered 1 h before the addition of 8 mg/ml urinary proteins for 2 h. Cell culture supernatant was subjected to DCFH-DA flow cytometry analysis of ROS levels (a, b). After treatment with urinary proteins for 4 h, aliquots of cell lysate were subjected to Western blot analysis of p-ERK and t-ERK level (c, d). (b) *F*(3 8) = 4.901; (d) *F*(5 12) = 5.581. ^∗^*P* < 0.05 and ^∗∗^*P* < 0.01. CON: control; NAC: N-acetylcysteine; CAT: catalase; UP: urinary proteins.

In this study, we first evaluated the activity of ERK pathway by examining the expression of phosphorylated ERK (p-ERK), a key marker of ERK pathway activation. As shown in Figure 1, the expression of p-ERK was significantly increased in TECs after treatment with urinary protein at 2 h, suggesting the activation of ERK pathway after urinary protein overload. Meanwhile, we found that the ROS production was remarkably elevated. Subsequently, to clarify the relationship between ERK pathway activation and ROS overproduction, we pretreated protein overload HK-2 cells with antioxidants (NAC and CAT). The results of flow cytometry and Western blot showed that the expression of p-ERK decreased with the application of antioxidants. Collectively, these results demonstrated that ERK pathway was activated in urinary-treated TECs and mediated by a ROS-dependent mechanism.

### 3.2. ERK Pathway Activation Was Involved in Urinary Protein-Induced TEC Injury

**Figure 2:**
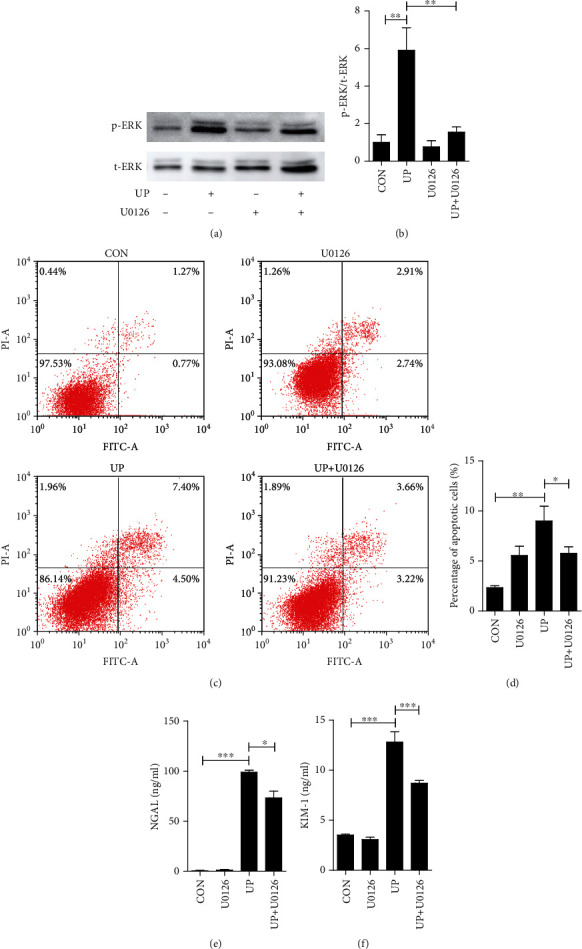
Effect of MEK inhibitor on urinary protein-induced injury. MEK inhibitor U0126 (10 *μ*M) was administered 1 h before the addition of 8 g/l urinary proteins. The aliquots of cell lysate were subjected to Western blot analysis of p-ERK and t-ERK levels (a, b), and cellular apoptosis was assessed by Annexin V-FITC/PI flow cytometry analysis (c, d) at 48 h. Cell culture supernatant was subjected to ELISA analysis of the NGAL and KIM-1 secretion at 12 h (e, f). (b) *F*(3 8) = 13.385; (d) *F*(3 8) = 8.539; (e) *F*(3 12) = 207.111; (f) *F*(3 16) = 72.157. ^∗^*P* < 0.05, ^∗∗^*P* < 0.01, and ^∗∗∗^*P* < 0.001. CON: control; UP: urinary proteins.

Since previous studies have shown that exposure to urinary proteins resulted in injury and even apoptosis in HK-2 cells, we then intended to further explore whether the ERK pathway was the responsible process. Firstly, the inhibiting effect of an ERK inhibitor U0126 on ERK pathway was tested by Western blot. As shown in Figures 2(a) and 2(b), pretreatment with U0126 could effectively suppress the expression of p-ERK, suggesting effective blockage of U0126 in activated ERK pathway. Then, we evaluated the early and late apoptosis by coupled staining with FITC annexin V and PI. The results showed that ERK inhibitor could attenuate cellular apoptosis induced by urinary proteins (Figures 2(c) and 2(d)). Furthermore, U0126 reduced the level of tubular injury markers NGAL and KIM-1, which have been increased with urinary protein stimulation (Figures 2(e) and 2(f)). These results indicated that ERK pathway activation caused by urinary protein led to cellular injury and apoptosis in HK-2 cells.

### 3.3. Autophagic Response Was Triggered by ERK Pathway after Exposure of TECs to Urinary Proteins

**Figure 3:**
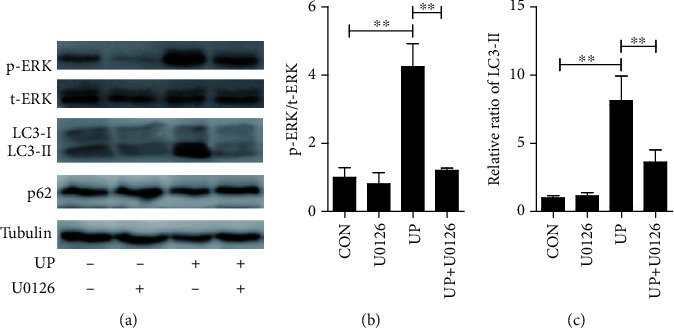
Effect of MEK inhibitor on urinary protein-induced autophagy. MEK inhibitor U0126 (10 *μ*M) was administered 1 h before the addition of 8 g/l urinary proteins for 8 h. Aliquots of cell lysate were subjected to Western blot analysis of p-ERK, t-ERK, LC3, and p62 levels. (b) *F*(3 12) = 16.329; (c) *F*(3 8) = 11.229. ^∗∗^*P* < 0.01. CON: control; UP: urinary proteins.

As our previous research has found that the cellular autophagy was activated by exposure to urinary proteins, the exact role of ERK pathway played in autophagy activation was further explored in this study. As shown in Figure 3, the amount of p62 had no significant change but the amount of LC3-II was remarkably increased after exposure to urinary protein for 8 h, indicating the activation of autophagy. However, these effects could be eliminated by suppressing the expression of p-ERK with U0126, suggesting that autophagy activation by urinary protein was definitely ERK pathway dependent.

### 3.4. Adaptive Autophagic Response Alleviated TEC Injury Induced by Urinary Proteins

**Figure 4:**
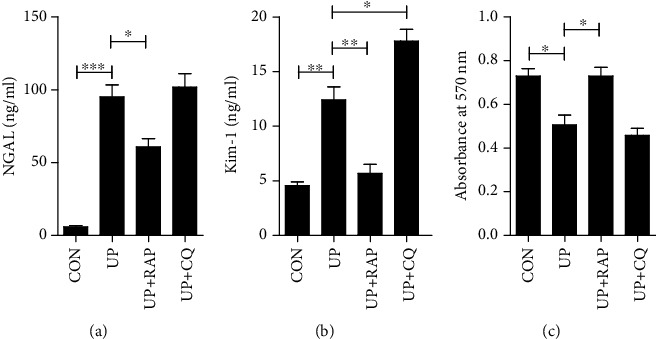
Effects of rapamycin or chloroquine on damage to TECs induced by urinary protein. (a, b) The levels of supernatant NGAL and KIM-1 were measured by ELISA after exposure to vehicle, urinary proteins (UP, 8 mg/ml), UP (8 mg/ml) plus rapamycin (RAP, 10 *μ*M), or UP (8 mg/ml) plus chloroquine (CQ, 10 *μ*M). (c) Cell proliferation was assessed by MTT assay as described in (a). ^∗^*P* < 0.05, ^∗∗^*P* < 0.01. CON: control; UP: urinary proteins; RAP: rapamycin; CQ: chloroquine.

We next evaluated the role of autophagy in TEC injury. It is well known that rapamycin increases autophagy flux, while chloroquine blocks autophagy pathway. Thus, whether rapamycin or chloroquine affected the urinary protein-induced cellular injury was examined. We found that increased NGAL and KIM-1 release (Figures 4(a) and 4(b)) and growth inhibition (Figure 4(c)) were reduced by rapamycin, while opposite results were obtained with chloroquine when assessing KIM-1 secretion. These findings indicated that autophagic response activated by ERK pathway was adaptive, for it could attenuate renal tubular injury caused by urinary proteins.

## 4. Discussion

In the present study, we demonstrated that ERK pathway was activated by a ROS-dependent mechanism in TEC cells exposed to urinary protein. What is more, it was proved to play dual roles in the cellular injury. On the one hand, it caused cellular injury directly; on the other hand, it could induce adaptive autophagic response to relieve the cellular injury. The complexity of the ROS-ERK pathway in TEC cells under condition of urinary protein overload was clarified for the first time.

ERK pathway is involved in various cellular processes [19]. In the present study, the results showed that once ERK activity was inhibited, urinary protein-induced TEC apoptosis and the release of factors that causes renal injury were reduced, suggesting the unfavorable role of ERK pathway in the process. However, the mechanism by which ERK pathway specifically mediates cell injury is currently unclear; it may be related to mitochondrial dysfunction [20, 21] or suppression of the Akt pathway which is positive to cell survival [22]. Notably, ERK inhibitor U0126 failed to completely block urinary protein-induced cell damage, suggesting that a non-ERK pathway may also be involved in mediating the toxicity of urinary proteins.

Previous studies have shown that urinary proteins could induce endoplasmic reticulum stress and mitochondrial dysfunction in TECs, resulting in massive ROS production [23, 24]. As an important intracellular messenger, ROS activates the ERK pathway via stimulation of EGF and PDGF receptors [25, 26] or by the way of oxidizing C118 residues in Ras directly [27]. Meanwhile, ROS could inhibit ERK-specific phosphatase activity and reduce the degradation of phosphorylated ERK [25]. Consistent with these studies, our study found that while urinary proteins stimulated large amounts of ROS generation, the ERK pathway of TECs was activated in TECs. Besides, inhibition dose of ROS in vitro, NAC, and CAT could block the activation of ERK. These results led us to conclude that oxidative stress induced by urinary protein is extremely significant for ERK pathway activation.

The ERK pathway has long been considered to be an important regulatory pathway for cellular autophagy [28]. Wang and Zeng have illustrated that ERK pathway could induce autophagy through Beclin 1 [29, 30] Therefore, we posited that ERK pathway was involved in autophagy behavior and confirmed this hypothesis through protein overload model of cultured TECs. Using experimental methods consistent with our previous studies, we found that the expression of LC3-II was significantly increased, and the expression of autophagic substrate p62 was decreased after ERK pathway activation. As p62 is selectively incorporated into autophagosomes and is efficiently degraded by autophagy, the decreased expression of p62 indicates that autophagy degradation is not affected by ERK pathway. Meanwhile, the accumulation of LC3-II suggests autophagy is activated [14]. However, the increased dose of LC3-II was reverted and p62 returned to normal levels under stimulation of U0126, which inhibited ERK pathway effectively. These results reveal that the activation of autophagy induced by urinary proteins is at least partially dependent on the ERK pathway. An increasing number of articles have confirmed the truth that increasing autophagy in cells influenced by pathogenic factors could partially combat cellular injury and increase the cell survival rate [31, 32]. Consistent with the previous studies, when autophagic flux was promoted by rapamycin, KIM-1 and NGAL excretion decreased despite of the increasing cell proliferation. Meanwhile, cellular injury tended to be aggravated when the autophagic pathway was blocked by chloroquine. Therefore, our results suggest that the activation of ERK pathway roused adaptive autophagic response under urinary protein stimulation.

Researchers reported that the apoptotic behavior of cells depend on the sustained activation of ERK within a certain period of time [33], and apoptosis could be aggravated when signals of activated ERK was delivered to the nucleus [34]. Specifically, the ERK activated messages in cytoplasm would induce autophagy [17]. Thus, we believe that when stimulated by urinary proteins, ERK pathway in the cytoplasm will activate an autophagy-related pathway and initiate an autophagy-associated adaptive response. However, under sustained stimulation of urinary proteins, the signals of activated ERK in the cytoplasm is likely to reenter the nucleus. This may attenuate autophagy and the ability of the cells to resist injury. In addition, the activated ERK in the nucleus will contact apoptosis-related pathways and result in cell apoptosis. In the absence of external interventions, persistent urinary proteins will eventually damage the TECs via the ERK pathway. In all, the results above suggest that the exact regulation of activation time and/or location of ERK pathway might offer a novel therapeutic opportunity in almost all the primary and secondary nephropathies with excessive urinary proteins, especially in diabetic nephropathy. However, the results of this study are still not enough to explain the role and mechanism of the ERK pathway in TECs. For example, scaffold proteins can accelerate the activation of this pathways and then participate in the regulation of the signal sustainability and intensity [35]. Hence, further information is required to explain the ERK pathway.

## 5. Conclusion

**Figure 5:**
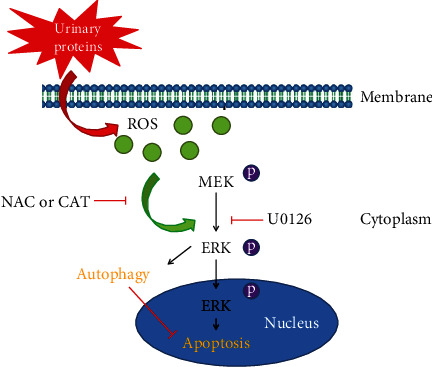
Schematic representation of the mechanisms of ROS-ERK pathway in urinary protein-induced injury in TECs. Urinary protein-induced ROS production triggers a cascade of phosphorylation reactions in the ERK pathway, which is a common mediator of protective autophagy and injury in TECs. In the early phase, cytoplasm p-ERK induces autophagy-associated adaptive response. Once stimulation is sustained, cytoplasm p-ERK transfers into the nucleus and elicits cell death.

In summary, our work suggests that the ROS-ERK pathway is a double-edged sword when stimulated by urinary proteins. It can induce TEC injury and in the meantime provide an autophagy-associated adaptive response (Figure 5). Thus, the accurate regulation of this pathway is likely to be a promising target for patients suffer from TEC injury induced by urinary proteins.

## Data Availability

The datasets used and/or analyzed during the current study are available from the corresponding author on reasonable request.

## Abbreviations

ERK: Extracellular signal-regulated protein kinases
KIM-1: Kidney injury molecule-1
NGAL: Neutrophil gelatinase-associated lipocalin
ROS: Cellular reactive oxygen species
TECs: Renal tubular epithelial cells
LC3: Microtubule-associated protein 1 light chain 3
NAC: N-acetylcysteine
CAT: Catalase
RAP: Rapamycin
CQ: Chloroquine
CON: Control
UP: Urinary proteins.
